# Effect of Aerobic Exercise Training on Blood Pressure in Indians: Systematic Review

**DOI:** 10.1155/2016/1370148

**Published:** 2016-07-17

**Authors:** Sonu Punia, Sivachidambaram Kulandaivelan, Varun Singh, Vandana Punia

**Affiliations:** ^1^Department of Physiotherapy, GJUST, Hisar 125001, India; ^2^Human Resource Development Center, GJUST, Hisar 125001, India

## Abstract

*Introduction*. High blood pressure (BP) is one of the most important modifiable risk factors for cardiovascular diseases, which accounts for one in every eight deaths worldwide. It has been predicted that, by 2020, there would be 111% increase in cardiovascular deaths in India. Aerobic exercise in the form of brisk walking, jogging, running, and cycling would result in reduction in BP. Many meta-analytical studies from western world confirm this. However, there is no such review from Indian subcontinent.* Objective*. Our objective is to systematically review and report the articles from India in aerobic exercise on blood pressure.* Methodology*. Study was done in March 2016 in Google Scholar using search terms “Aerobic exercise” AND “Training” AND “Blood pressure” AND “India.” This search produced 3210 titles.* Results*. 24 articles were identified for this review based on inclusion and exclusion criteria. Total of 1107 subjects participated with median of 25 subjects. Studies vary in duration from +3 weeks to 12 months with each session lasting 15–60 minutes and frequency varies from 3 to 8 times/week. The results suggest that there was mean reduction of −05.00 mmHg in SBP and −03.09 mmHg in DBP after aerobic training.* Conclusion*. Aerobic training reduces the blood pressure in Indians.

## 1. Introduction

Elevated blood pressure, also known as hypertension, is one of the most important modifiable risk factors for cardiovascular disease and is accounting for 10% of overall deaths in India [1]. In a meta-analysis of 142 studies from rural and urban populations of India, it was concluded that hypertension is emerging as a major health problem in India [2] and is more in urban than in rural subjects [3] and is associated with a higher risk of developing coronary heart disease (CHD), congestive heart failure, and stroke [4, 5].

Aerobic exercises are advised for health promotion and prophylaxis for many cardiovascular diseases. They refer to all exercises that involve major muscle groups and improve oxygen consumption by the body. Many methods of aerobic exercise are available like walking, jogging, running, cycling, and others. Recent meta-analytical study from western world confirms that aerobic exercise would result in clinically significant reduction in blood pressure [6]. Aerobic exercise such as walking not only improves fitness but also improves overall quality of life and decreases all-cause mortality [7, 8].

Even though it is confirmed from western world that exercises are helpful, there is little available evidence from India. Many of Indian literatures are grey in nature, that is, short studies done for postdoctoral dissertation or doctoral thesis which are not available in standard electronic database, namely, Pubmed, CINHAL, and EMBASE. However, recent rise in electronic journals with or without print copy encourages young researchers from India to publish their research in them. But these journals are not indexed in major databases and hence are not noticed and reported by other authors. Thus the objective of this paper is to collect the available literature from India using secondary search engine “Google Scholar” and derive the conclusion whether aerobic exercise is reducing blood pressure in normal and pathological conditions or not in Indians.

## 2. Methodology

Computerized literature search was performed using “Google Scholar” search engine during March 2016. Keywords or search protocol used for this review is “Aerobic Exercise” AND “Training” AND “Blood Pressure” AND “India.” Inclusion criteria for this review were as follows: (i) experimental study with post-premeasurement of BP, (ii) aerobic exercise as the exercise intervention with minimal duration of 3 weeks, (iii) full-text articles in English published in journals with ISSN number, (iv) study done in any part of India without any time limitation, and (v) subjects being either normal or with following clinical conditions: obesity, hypertension, and diabetes. A main exclusion criterion was other clinical conditions (2 articles were excluded based on this criterion). Search protocol resulted in 3210 articles. Authors Sonu Punia and Sivachidambaram Kulandaivelan reviewed 3210 titles individually and selected articles based on inclusion and exclusion criteria. Sonu Punia selected 19 articles and Sivachidambaram Kulandaivelan selected 24 articles. Authors Vandana Punia and Varun Singh studied 43 articles and selected 25 articles (18 were duplicates). Results were prepared by Varun Singh. During this, we found two articles which used the same results in their papers [9, 10]. Recent one was excluded from the review [9].

Mean difference with 95% CI (confidence interval) for individual studies was calculated using mean, standard deviation (SD), and sample size (this was not possible in [11–15] due to nonavailability of SD). If there are 2 or more groups in an article [12, 15–17], standard mean of all groups BP values was used as single measure. Subtotal and overall reduction in SBP and DBP were calculated by adding individual BP values divided by number of studies ([18] was excluded from analysis due to this criterion). Mean difference with 95% CI was calculated using standard formula as described earlier.

## 3. Results

We selected 24 articles [10–33] with 1107 subjects (mean: 46; median: 25) for this review. Even though we did not use any time limits, all articles included in this reviews were from 2009 to 2015. Out of 24 articles, 13 articles [10–13, 16, 19–26] used healthy subjects and 11 articles [14, 15, 17, 18, 27–33] used clinical condition subjects with hypertension [14, 18, 27, 28] and diabetes [15, 17, 29–33]. Aerobic exercise was performed for the mean period of 40 minutes (median: 30) with a frequency of 4.8 days·week^−1^ (median: 5) and total duration of 12.5 weeks (median: 12) (Table 1).

A total of 5 articles [11, 16, 19–21] were included in young healthy adults population with total of 165 participants (mean: 33; median: 15; males/females: 73/92). The number of sessions in a week ranged from 3 to 8 sessions (mean: 5; median: 4). Total duration of program ranged from 4 to 16 weeks (mean: 11.2; median: 12.0). A total of 6 articles [12, 13, 22–25] were included in healthy middle and old age population with total of 541 participants (mean: 90; median: 25). Four articles mentioned the duration of session which ranged from 15 minutes to 45 minutes (mean: 35; median: 30). The number of sessions in a week ranged from 3 to 6 sessions (mean: 4.7; median: 5). Total duration of program ranged from 3 to 16 weeks (mean: 9; median: 11). A total of 2 articles [10, 26] were included in obese adults population with total of 40 participants (mean: 20; male/female: 30/10) (Table 1).

A total of 4 articles [14, 18, 27, 28] were included in aerobic exercise in hypertensive patients population with total of 122 participants (mean: 30.5; median: 28.5). Duration of exercise in single session ranged from 30 minutes to 60 minutes (mean: 40; median: 40). Frequency of exercise per week ranged from 3 to 5 days (mean: 4; median: 4). Total duration of exercise program ranged from 6 to 8 weeks (mean: 6.5; median: 6). A total of 7 articles [15, 17, 29–33] were included in aerobic exercise in diabetic patients population with total of 239 participants (mean: 34; median: 24). Duration of single session ranged from 15 minutes to 45 minutes (mean: 34; median: 30). Frequency of exercise per week ranged from 3 days to 7 days (mean: 5.3; median: 6). Total duration of the program ranged from 7 weeks to 52 weeks (mean: 21; median: 16) (Table 1).

Table 2 shows the effect of aerobic training of 3 weeks or more on blood pressure in individual studies basis. The results are classified into healthy adults and other clinical conditions, that is, hypertension and diabetes.

**Figure 1:**
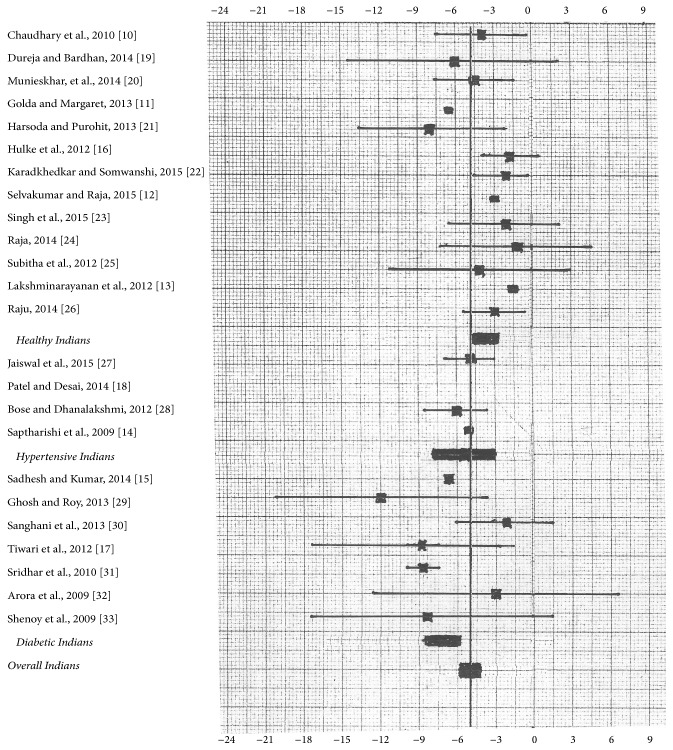
Summary of aerobic training on SBP (mean difference with 95% CI) in Indians. Bold vertical line is meta-analysis mean (*n* = 23).

Figure 1 shows summary of aerobic training on SBP in Indians. There is a mean reduction of 3.71 mmHg (95% CI: −2.74 to −4.68) in healthy Indians, 5.38 mmHg (95% CI: −2.99 to −7.77) in hypertensive Indians, and 7.24 mmHg (95% CI: −5.89 to −8.59) in diabetic Indians. Overall, aerobic training with mean duration of 12.5 weeks reduced SBP to 05.00 mmHg (95% CI: −4.18 to −5.82) in Indians.

**Figure 2:**
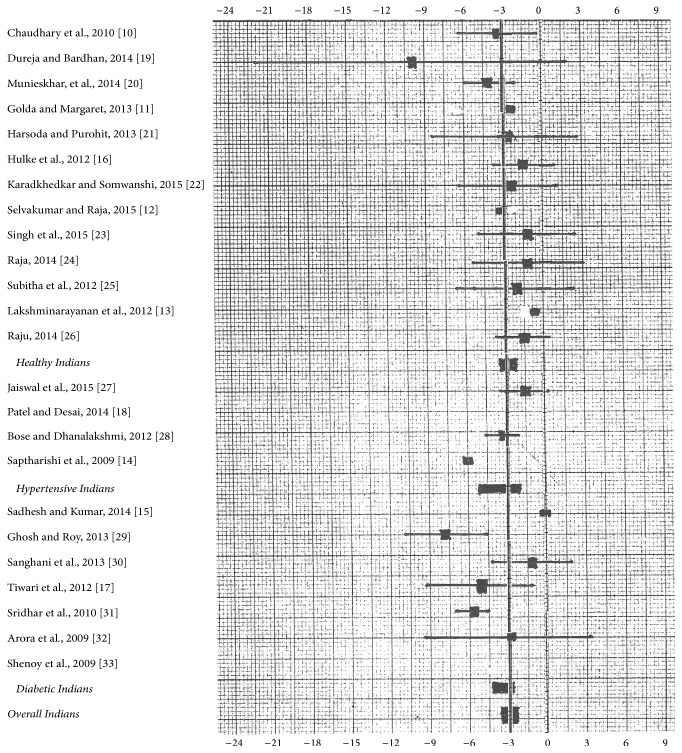
Summary of aerobic training on DBP (mean difference with 95% CI) in Indians. Bold vertical line is meta-analysis mean (*n* = 23).

Figure 2 shows summary of aerobic training on DBP in Indians. There is a mean reduction of 2.79 mmHg (95% CI: −2.12 to −3.46) in healthy Indians, 3.66 mmHg (95% CI: −2.02 to −5.30) in hypertensive Indians, and 3.41 (95% CI: −2.61 to −4.21) in diabetic Indians. Overall, aerobic training with mean single session duration of 40 minutes reduced DBP to 3.09 mmHg (95% CI: −2.57 to −3.61) in Indians.

Out of 24 included articles (23 for meta-analysis), 10 studies used control group (5 healthy subjects and 5 subjects with clinical condition) [10, 12, 14, 17, 23, 24, 26, 30, 32, 33]. Subgroup analysis of control versus experimental group in healthy Indian subjects showed mean reduction of 2.92 mmHg in SBP and 2.25 mmHg in DBP in favor of experimental group. The same analysis in clinical condition showed mean reduction of 5.4 mmHg in SBP and 2.6 mmHg in DBP in favor of experimental group.

## 4. Discussion

Primary objective of this review is to collect and present the literature from India of aerobic exercise on blood pressure. We found 24 articles from different parts of India (north, 5, south, 10, east, 3, and west, 6). Results show 5.00 mmHg reduction in SBP and 3.09 mmHg reduction in DBP. This result is supported by several meta-analyses from western population [6, 34–36]. Cornelissen and Smart [6] studied the effect of aerobic exercise on blood pressure in 105 study groups (93 RCTs) with 3500 subjects. Their results showed mean difference (MD) of −3.5 mmHg in SBP and −2.5 mmHg in DBP. Whelton et al. [35] studied effect of aerobic exercise on blood pressure in 53 RCTs (2419 subjects). They found MD of −3.84 mmHg in SBP (−4.97 to −2.72) and −2.58 mmHg in DBP (−3.35 to −1.81). Halbert et al. [36] analyzed 26 RCTs of at least 4-week aerobic training on blood pressure. Their results are similar to our findings (SBP, −04.70 mmHg, and DBP, −03.10 mmHg). All the three meta-analyses included here used either normotensive or hypertensive subjects only; diabetes was not reported in these meta-analyses. So we did another subanalysis for healthy and hypertensive studies which showed mean reduction of 4.03 mmHg (95% CI: −3.05 to −5.01) in SBP and of 2.96 mmHg (95% CI: −2.29 to −3.63) in DBP.

As compared to control group, aerobic training reduced 4.16 mmHg in SBP and 2.43 mmHg in DBP in experimental group of Indians which is lower end of 95% CI in post-premean MD of present study. This is in agreement with western meta-analysis [6, 35, 36]. We compared individual studies' SBP MD (95% CI) with overall meta-analysis' SBP mean (Figure 1) and results showed that 16 of 23 studies are in agreement with meta-analysis' SBP mean. Three articles (2 healthy subjects [16, 22] and 1 diabetic subject [31]) only deviated from meta-analysis' SBP mean. 17 of 23 studies' DBP MD (95% CI) are in agreement with meta-analysis' DBP mean (Figure 2). Two articles (both of diabetic population [29, 31]) only deviated from meta-analysis' DBP mean. This suggests that all articles' results are reliable to each other. The mean reduction of BP in all the three western meta-analyses [6, 35, 36] is within 95% CI of present meta-analysis; hence, we could predict that future studies from India will reproduce the same reduction after aerobic training.

Hypertension is a growing problem in India with every third to fifth Indian as hypertensive [2, 37, 38]. Approximately 10% of death in India was attributable to high blood pressure [39]. It is the third leading risk factor for disease burden in both developed and developing nations worldwide [40]. According to SEEK study, ischemic heart disease, stroke, and peripheral vascular diseases are significantly higher in hypertensive Indian population than in control [41]. In a recent meta-analysis of 18 prospective cohorts, compared to normotension, prehypertension elevated the risk of cardiovascular disease (CVD) by 1.55 (relative risk, RR), coronary heart disease (CHD) by 1.50, and stroke by 1.71 [4]. It is one of the most common modifiable risk factors in CVD. Mean reduction of 3.0 mmHg in SBP could reduce mortality from CHD by 6% and from stroke by 9% [42]. Another study found that rise of SBP by 3.0 mmHg and DBP by 2.3 mmHg would result in estimated 12% increased risk for CHD and 24% increased risk for stroke [43].

There are some limitations observed in this review. We used improvement in BP using after minus before values, except one study [12], and one study used MD instead of blood pressure values [18]. Sample size, training duration, session duration, and frequency of training are varied between studies which may underestimate or overestimate our interpretations in results and we do not use any statistical tests to see their association with BP lowering effect.

## 5. Conclusion

Aerobic training of 4-week duration would reduce BP to a clinically significant level in Indian population. This reduction is more pronounced in clinical conditions like hypertension and diabetes. So this type of training should be used as primary prevention in high risk population and as secondary prevention in hypertension, diabetes, and so forth.

## Figures and Tables

**Table 1:** Basic characteristics of included studies.

Author et al., year	Population (male/female)	Intensity	Session duration	Frequency	Total duration
Healthy young adults (*n* = 165)					
Dureja and Bardhan, 2014 [19]	05 (05/00)	7–10 Km/hr	15–25 minutes	6 days/week	4 weeks
Munieskhar et al., 2014 [20]	50 (00/50)	NA	30 minutes	7 days/week	12 weeks
Golda and Margaret, 2013 [11]	10 (10/00)	65–80% HRR	90 minutes	3 days/week	12 weeks
Harsoda and Purohit, 2013 [21]	15 (15/00)	10 Km/hr	30 minutes	5 days/week	12 weeks
Hulke et al., 2012 [16]	85 (43/42)	RPE	60 minutes	8 sessions/week	16 weeks

Middle and old age (*n* = 541)					
Karadkhedkar and Somwanshi, 2015 [22]	30 (00/30)	60–75% HR_max_	30 minutes	5 days/week	16 weeks
Selvakumar and Raja, 2015 [12]	20	NA	NA	5 days/week	12 weeks
Singh et al., 2015 [23]	17 (06/11)	50–75% APMHR	30 minutes	5 days/week	3 weeks
Raja, 2014 [24]	15	NA	NA	6 days/week	13 weeks
Subitha et al., 2012 [25]	38 (18/20)	NA	15 minutes	3 days/week	10 weeks
Lakshminarayanan et al., 2012 [13]	421	Brisk	30 minutes	4 days/week	10 weeks

Obese adults (*n* = 40)					
Chaudhary et al., 2010 [10]	10 (00/10)	60–70% MHR	Till exhaustion	3 days/week	6 weeks
Raju, 2014 [26]	30 (30/00)	Varying	60 minutes	6 days/week	12 weeks

Hypertensive adults (*n* = 122)					
Jaiswal et al., 2015 [27]	15	50–80% HRR	30 minutes	5 days/week	6 weeks
Patel and Desai, 2014 [18]	30 (21/09)	NA	30 minutes	NA	6 weeks
Bose and Dhanalakshmi, 2012 [28]	50	60–75% HR_max_	50 minutes	3 days/week	6 weeks
Saptharishi et al., 2009 [14]	27 (19/08)	Brisk	50–60 minutes	4 days/week	8 weeks

Diabetic adults (*n* = 239)					
Sadhesh and Kumar, 2014 [15]	15	NA	45 minutes	7 days/week	7 weeks
Ghosh and Roy, 2013 [29]	24 (00/24)	65–75% run/walk	15 minutes	6 days/week	25 weeks
Sanghani et al., 2013 [30]	74	Varying	45 minutes	6 days/week	26 weeks
Tiwari et al., 2012 [17]	51 (28/23)	NA	30 minutes	NA	12 weeks
Sridhar et al., 2010 [31]	55 (30/25)	NA	45 minutes	7 days/week	52 weeks
Arora et al., 2009 [32]	10 (06/04)	NA	30 minutes	3 days/week	8 weeks
Shenoy et al., 2009 [33]	10 (06/04)	NA	30 minutes	3 days/week	16 weeks

APHRM, age predicted heart rate maximum; HR_max_, heart rate maximum; HRR, heart rate reserve; MHR, maximal heart rate; NA, not available; RPE, ratings of perceived exertion.

**Table 2:** Effect of aerobic exercise training on blood pressure in Indians.

Author et al., year	Before SBP	After SBP	Before DBP	After DBP	Mean difference (MD)
Healthy young adults (*n* = 165)					
Dureja and Bardhan, 2014 [19]	116.00 ± 5.47	110.00 ± 6.07	79.00 ± 8.94	69.00 ± 7.41	SBP −06.00 DBP −10.00
Munieskhar et al., 2014 [20]	99.6 ± 8.38	95.1 ± 6.9	66.2 ± 5.67	62.15 ± 3.43	SBP −04.50 DBP −04.05
Golda and Margaret, 2013 [11]	128.5	121.7	80.52	78.27	SBP −06.80 DBP −02.25
Harsoda and Purohit, 2013 [21]	120.2 ± 7.55	112.2 ± 6.88	78.4 ± 8.01	75.6 ± 6.73	SBP −08.00 DBP −02.80
Hulke et al., 2012 [16]	113.74 ± 8.36	112.36 ± 6.16	73.72 ± 8.4	72.29 ± 6.56	SBP −01.38 DBP −01.43

Middle and old age (*n* = 541)					
Karadkhedkar and Somwanshi, 2015 [22]	126.46 ± 6.30	124.39 ± 1.86	86.03 ± 7.12	83.60 ± 7.57	SBP 02.07 DBP 02.43
Selvakumar and Raja, 2015 [12]	Control 134.34	131.48	Control 90.18	86.96	SBP −02.86 DBP −03.22
Singh et al., 2015 [23]	120.3 ± 6.6	118.1 ± 6.0	82.7 ± 6.3	81.5 ± 3.6	SBP −02.20 DBP −01.20
Raja, 2014 [24]	129.87 ± 7.96	128.60 ± 7.92	85.60 ± 5.65	84.33 ± 5.51	SBP −01.27 DBP −01.27
Subitha et al., 2012 [25]	126.89 ± 16.9	122.71 ± 14.4	77.84 ± 10.5	75.82 ± 9.46	SBP −04.18 DBP −02.02
Lakshminarayanan et al., 2012 [13]	122.40	120.84	76.81	76.07	SBP −01.56 DBP −00.74

Obese Adults (*n* = 40)					
Chaudhary et al., 2010 [10]	128.10 ± 4.95	124.20 ± 2.82	85.00 ± 3.27	81.80 ± 3.12	SBP −03.90 DBP −03.20
Raju, 2014 [26]	131.03 ± 4.642	127.93 ± 4.89	84.00 ± 4.02	82.33 ± 3.88	SBP −03.10 DBP −01.67

Hypertensive adults (*n* = 122)					
Jaiswal et al., 2015 [27]	129.46 ± 2.87	124.6 ± 1.95	81.37 ± 2.37	79.86 ± 2.56	SBP −04.86 DBP −01.51
Patel and Desai, 2014 [18]					SBP −03.35 DBP −02.00
Bose and Dhanalakshmi, 2012 [28]	145.87 ± 5.73	139.87 ± 5.92	94.80 ± 2.86	91.33 ± 2.59	SBP −06.00 DBP −03.47
Saptharishi et al., 2009 [14]	128.6	123.3	87.4	81.4	SBP −05.30 DBP −06.00

Diabetic adults (*n* = 239)					
Sadhesh and Kumar, 2014 [15]	119.69	112.89	74.45	74.45	SBP −06.80 DBP 00.00
Ghosh and Roy, 2013 [29]	139.66 ± 14.38	127.83 ± 13.9	80.00 ± 5.57	72.25 ± 5.46	SBP −11.80 DBP −07.75
Sanghani et al., 2013 [30]	133.47 ± 13.23	131.20 ± 10.36	84.29 ± 9.38	83.24 ± 9.02	SBP −02.27 DBP −01.05
Tiwari et al., 2012 [17]	131.92 ± 18.25	121.84 ± 15.03	84.76 ± 12.12	79.50 ± 7.48	SBP −10.08 DBP −05.26
Sridhar et al., 2010 [31]	144.24 ± 2.87	135.53 ± 3.54	88.59 ± 3.92	82.82 ± 1.07	SBP −08.71 DBP −05.77
Arora et al., 2009 [32]	132 ± 8.5	124 ± 11.6	84 ± 5.3	81 ± 8.2	SBP −08.00 DBP −03.00
Shenoy et al., 2009 [33]	132 ± 8.5	129 ± 11.6	84 ± 5.3	83 ± 7.4	SBP −03.00 DBP −01.00
